# Study on the Isolation of Two Atrazine-Degrading Bacteria and the Development of a Microbial Agent

**DOI:** 10.3390/microorganisms7030080

**Published:** 2019-03-14

**Authors:** Jiangwei Zhu, Li Fu, Caihua Jin, Zili Meng, Ning Yang

**Affiliations:** 1 Collaborative Innovation Center of Sustainable Forestry in Southern China of Jiangsu Province, (Nanjing Forestry University), Nanjing 210037, China; 2 College of Materials and Environmental Engineering, Hangzhou Dianzi University, Hangzhou 310018, China; fuli_hdu@163.com; 3 Pudong Agrotechnology Extension Center, Shanghai 201201, China; jch_jchua@163.com; 4 Shangqiu Academy of Agricultural and Forestry Sciences, Shangqiu 476000, China; gousi2012@126.com; 5 Ji’nan Landscape Flower and Plantlet Breeding Center, Jinan 250103, China; yang_nn@hotmail.com

**Keywords:** atrazine, biodegradation, *Bacillus licheniformis*, *Bacillus megaterium*, microbial consortium

## Abstract

Two bacteria capable of efficiently degrading atrazine were isolated from soil, and named ATLJ-5 and ATLJ-11. ATLJ-5 and ATLJ-11 were identified as *Bacillus licheniformis* and *Bacillus megaterium*, respectively. The degradation efficiency of atrazine (50 mg/L) by strain ATLJ-5 can reach about 98.6% after 7 days, and strain ATLJ-11 can reach 99.6% under the same conditions. The degradation of atrazine is faster when two strains are used in combination. Adding the proper amount of fresh soil during the degradation of atrazine by these two strains can also increase the degradation efficiency. The strains ATLJ-5 and ATLJ-11 have high tolerance to atrazine, and can tolerate at least 1000 mg/L of atrazine. In addition, the strains ATLJ-5 and ATLJ-11 have been successfully made into a microbial agent that can be used to treat atrazine residues in soil. The degradation efficiency of atrazine (50 mg/kg) could reach 99.0% by this microbial agent after 7 days. These results suggest that the strains ATLJ-5 and ATLJ-11 can be used for the treatment of atrazine pollution.

## 1. Introduction

Atrazine (2-chloro-4-ethylamino-6-isopropylamino-s-triazine) is used for weed control in crops such as maize, sugarcane, and citrus throughout the world [1]. The widespread and long-term use of atrazine results in high residue levels of atrazine in soil, which further causes water contamination [2]. Atrazine is considered as an endocrine disruptor and is potentially carcinogenic [3]. There are some negative impacts on plant photosynthesis as well as microorganism and human health [4,5,6]. Atrazine can inhibit the Hill reaction by binding to tyrosinase; for example, sublethal atrazine concentrations can induce a general inhibition on the growth and photosynthesis of *Chlorella kessleri*. In addition, it is considered a moderately persistent chemical in the environment as it has a long half-life, and greater mobility in soils than some other herbicides such as dipropetryne and simetryn [7]. Atrazine, due to its high toxicity, persistence, and mobility in the environment [8,9], was banned by the European Union in 2003, but it is still one of the most widely used herbicides against broad leaf weeds today in many countries, for example, the USA, Brazil, and Argentina [10]. Therefore, for a safe environment, the rapid elimination of atrazine from the site of contamination is considered to be of vital importance. An ideal method for treating atrazine residue is the use of microorganisms, as it is more cost-effective and environmentally friendly [11].

Microorganisms are of great importance in environmental cleaning and herbicide degradation. In microbially active soil, the mean half-life of atrazine is 2.4 times lower than that in sterile soil [12]. Many strains have been isolated and studied for their abilities in atrazine mineralization including members of the genera *Pseudomonas*, *Agrobacterium*, *Arthrobacter*, and *Norcardioides* [13,14,15]. However, the degradation efficiencies of the bacteria are not high enough in most cases, and there is also a lack of commercial microbial agents for the treatment of atrazine pollution. Therefore, it is still necessary to isolate highly effective atrazine-degrading bacteria and develop microbial agents in order to remove atrazine from atrazine-contaminated environments. In this study, two bacteria that could efficiently degrade atrazine were isolated from soil—*Bacillus licheniformis* ATLJ-5 and *Bacillus megaterium* ATLJ-11. In addition, the strains ATLJ-5 and ATLJ-11 have been successfully made into a microbial agent that is easy to commercialize and can be used to treat atrazine residues in soil. The microbial agent containing the strains ATLJ-5 and ATLJ-11 achieved good application results in this experiment.

## 2. Materials and Methods

### 2.1. Media

(1) The mineral salt medium consisted of (NH_4_)_2_SO_4_ 0.1 g, K_2_HPO_4_ 0.1 g, CaSO_4_ 0.05 g, MgSO_4_·7H_2_O 0.2 g, FeSO_4_·7H_2_O 0.01 g, and distilled water 1.0 L, pH 7.0. When making the mineral salt culture plate, 12 g agar powder was added to 1000 mL culture medium. (2) The isolation medium consisted of a mineral salt medium and 50 mg/L atrazine (it was the final concentration in the media) as the sole carbon source. (3) The enrichment medium consisted of peptone 6.5 g, yeast extract powder 5.5 g, beef extract powder 4.5 g, sodium chloride 5 g, and water 1.0 L, pH 7.0. (4) The nutrient broth medium consisted of peptone 10 g, beef extract powder 3 g, sodium chloride 5 g, and water 1.0 L, pH 7.2.

### 2.2. Determination of Atrazine and Its Metabolites

All determinations of atrazine and its metabolites in various samples were performed as follows. The samples were collected at regular intervals. Liquid samples were centrifuged to obtain cell-free supernatant and subjected to HPLC-MS/MS analysis. The soil samples were processed as follows: Using a stopper, 5 g of soil sample was transferred to a measuring cylinder and then 5 mL of distilled water was added and mixed well. Then 3 g NaCl and 20 mL acetonitrile were sequentially added, shaken vigorously for 5 min, and left to sit for 30 min. Next, 8 mL of acetonitrile layer was dried in a nitrogen stream until near dry and diluted to 2 mL for HPLC-MS/MS analysis [16,17].

The cell-free culture liquids and soil extracts were analyzed by HPLC-MS/MS on a 1260HPLC-6430 Triple quadrupole mass spectrometer (Agilent™, Santa Clara, CA, USA). Atrazine and metabolites were separated on an Agilent™ C18 column (3.0 × 100 mm, 1.8-Micron, P.N.959758-302) with a flow rate of 0.3 mL/min. Solutions of 0.1% formic acid–water (A) and acetonitrile (B) were used for HPLC gradient elution. The program of gradient elution was 0 to 0.5 min, 60% A; 2 to 9 min, 10% A; and 9.1 to 10 min, 60% A. The MS/MS analysis was performed in positive ESI (electrospray ionization) mode and transitions were measured by MRM (multiple reaction monitoring). Main parameters of MS/MS: atrazine (precursor ion: 216, product ion: 174/132, fragmenter: 132, CE: 13/21); hydroxyatrazine (precursor ion: 198, product ion: 156/86, fragmenter: 115, CE: 12/20); N-isopropylammelide (precursor ion: 171, product ion: 104/68, fragmenter: 120, CE: 15/15).

### 2.3. Isolation and Identification of Atrazine-Degrading Bacteria

The soil samples were collected from woodland in Nanjing City, China, where atrazine has been used for more than 10 years. The 5.0 g soil sample was mixed into a 100 mL isolation medium for shaking culture at 30 °C (100 rpm). The concentration of atrazine in the medium was determined every 24 h. The 5 mL culture solution with a 3-day degradation efficiency >60% was transferred to the enrichment medium containing 50 mg/L atrazine (the results of previous investigations indicated that the highest value of atrazine residue in soil or water was about 50 mg/L or 50 mg/kg), and the culture was continuously passaged more than five times. After the degradation efficiency was verified again, the above enrichment culture solution was smeared on the mineral salt culture plate containing atrazine for inverted culture at 30 °C for 72 h. The eugonic bacterial colonies were selected and repeatedly streaked on the culture plate to obtain the pure culture. Bacterial biochemical experiments were carried out using the method described by Dong Xiuzhu [18]. The new isolates were identified by using 16 SrRNA sequence analysis, and a phylogenetic tree was built.

Total DNA was extracted following the standard procedure described in the literature [19]. Primers 27F (5′-AGAGTTTGATCCTGGTCAG-3′) and 1492R (5′-TACGGCTACCTTGTTACGACT-3′) were used to amplify the 16S rDNA [20]. The PCR reaction was performed using a PCR instrument with the following cyclic procedures: Initial denaturation at 94 °C for 5 min, 35 cycles of denaturation at 94 °C for 1 min, annealing at 55 °C for 1 min, extension at 72 °C for 2 min, and final extension at 72 °C for 10 min [21]. The determined 16S rRNA gene (partial sequence) was aligned with those available in the GenBank database. A phylogenetic tree was made using ClustalX1.83 and MEGA 5.2 [22].

### 2.4. Preparation of Bacterial Inocula

The atrazine-degrading strains *B. licheniformis* ATLJ-5 and *B. megaterium* ATLJ-11 were isolated from soil. Prior to microcosm experiments, the bacteria were grown in a nutrient broth medium containing 50 mg/L atrazine at 30 °C (100 rpm). Cells were collected after overnight culture by centrifugation and washed with a sterilized saline. The cells were resuspended in saline to an approximate density of 2 × 10^5^ cells/mL. This bacterial suspension was stored to be used as inoculum for subsequent experiments.

### 2.5. Degradation of Atrazine by Strains ATLJ-5 and ATLJ-11

First, 5 mL of strain ATLJ-5 inoculum (2 × 10^5^ cells/mL) was inoculated in a 100 mL mineral salt culture medium containing atrazine (the final concentration of atrazine was 50 mg/L), and then incubated at 30 °C (100 rpm). The concentration of atrazine was determined every 24 h. The strain ATLJ-11 was tested according to the same procedure.

Then, 2.5 mL of strain ATLJ-5 inoculum (2 × 10^5^ cells/mL) and 2.5 mL of ATLJ-11 inoculum (2 × 10^5^ cells/mL) were simultaneously inoculated into mineral salt medium containing atrazine (the final concentration of atrazine was 50 mg/L), then incubated at 30 °C (100 rpm). The concentration of atrazine was determined regularly.

### 2.6. The Effect of Atrazine Concentration on the Growth of Bacteria

The concentration of atrazine in mineral salt medium was adjusted to 50 mg/L, 100 mg/L, 200 mg/L, 500 mg/L, and 1000 mg/L and then the strain ATLJ-5 was inoculated for shaking cultivation at 30 °C. The OD_600_ was determined regularly, so as to evaluate the tolerance of bacteria to atrazine. The strain ATLJ-11 was tested according to the same procedure.

### 2.7. Construction of a Simple Microbial Consortium and Development of a Microbial Agent

Construction of a simple microbial consortium: The enrichment medium (100 mL) was simultaneously inoculated with 2 mL of strain ATLJ-5 inoculum and 2 mL of strain ATLJ-11 inoculum, then incubated for 6 h at 30 °C (100 rpm), and finally stored at 0 °C for subsequent experiments.

A mineral salt medium containing atrazine (50 mg/L) was divided into three groups (*n* = 5). ① Add 2% (*v*/*v*) of the above microbial consortium to 200 mL of mineral salt medium containing atrazine (50 mg/L), while adding natural soil (add 2 g soil per 100 mL medium). ② Add only 2% (*v*/*v*) of the above microbial consortium. ③ Control group (abiotic control). Finally, all the above three groups were incubated at 30 °C, and the concentration of atrazine was determined regularly.

Preparation steps for the microbial agent containing strains ATLJ-5 and ATLJ-11: The strain ATLJ-5 inoculum, the strain ATLJ-11 inoculum, and the sterile nutrient broth medium were mixed (2:2:6, *v*/*v*); the mixture was incubated for 16 h at 30 °C. A mixture of rice bran powder and fresh soil (9:1, *w*/*w*, the soil used was consistent with the soil from which the atrazine-degrading bacteria were isolated) was used as a carrier and a 25 mL ready-made mixture was mixed with each 100 g carrier. The mixture was incubated at 30 °C for 12 h, during which time it was flipped every 4 h, then put into a sterile glass bottle for preservation at 0 °C.

Degradation of atrazine in soil by microbial agent: Atrazine was added into fresh soil with a concentration of 50 mg/kg, then the soil was put into 30 cm × 30 cm plastic boxes, forming a 10 cm depth in each box so as to make sure each box contained the same weight of soil (10 kg). The microbial agent was added into the soil (*n* = 5) and 0.5 g microbial agent was mixed into 30 mL of sterile water and then sprinkled on the soil in each box. For the control group 30 mL of sterile water was sprinkled. Then they were incubated at 30 °C, agitated every 6 h, and supplied with water properly. The concentration of atrazine in the soil was determined regularly.

## 3. Results and Discussion

### 3.1. Characterization of Atrazine-Degrading Bacteria

Two bacteria that could efficiently degrade atrazine were isolated from the soil and they were named ATLJ-5 and ATLJ-11. ATLJ-5 was a straight bacillus, 0.7–0.8 × 2.0–2.5 µm in size, G+, motile, facultative anaerobe, subterminal or middle spore, and formed opaque and rough colonies on the nutrient agar plate. It was positive in tests for catalase, Voges–Proskauer (VP test), gelatin liquefaction, methyl red test, and starch hydrolysis, but negative for indole test [23,24]. ATLJ-5 was identified as *B. licheniformis* according to 16S sequence analysis (GenBank accession number MH879786). 

**Figure 1:**
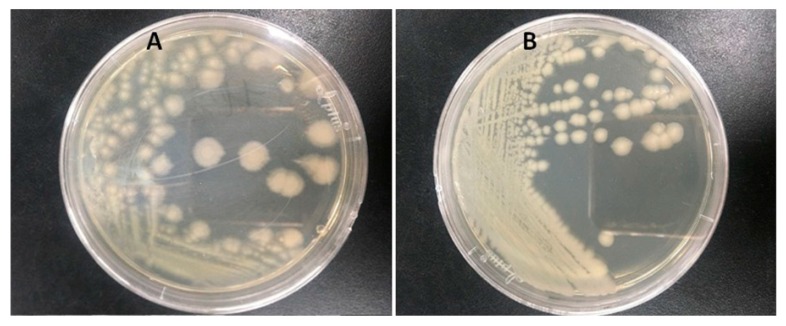
Bacterial colony photos of two atrazine-degrading bacteria. (**A**: strain ATLJ-5, **B**: strain ATLJ-11).

**Figure 2:**
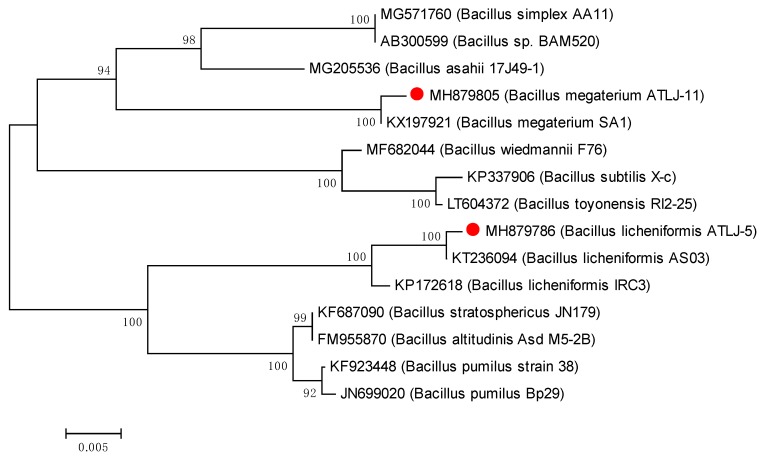
Phylogenetic tree of strain ATLJ-5 and strain ATLJ-11.

The strain ATLJ-11 was a straight or curvulate bacillus, G+, aerobiotic, mobile, spore-forming, 0.6–0.8 × 2.5–3.0 µm in size, and it formed opaque white rough colonies on the nutrient agar plate. It was positive for catalase, oxidase, gelatin liquefaction, starch hydrolysis, and VP test, but negative for methyl red test and indole test. ATLJ-11 was identified as *B. megaterium* (GenBank accession number MH879805). Bacterial colony photos of strain ATLJ-5 and strain ATLJ-11 were shown in Figure 1, and the phylogenetic tree was shown in Figure 2.

### 3.2. Degradation of Atrazine by Strains ATLJ-5 and ATLJ-11

**Figure 3:**
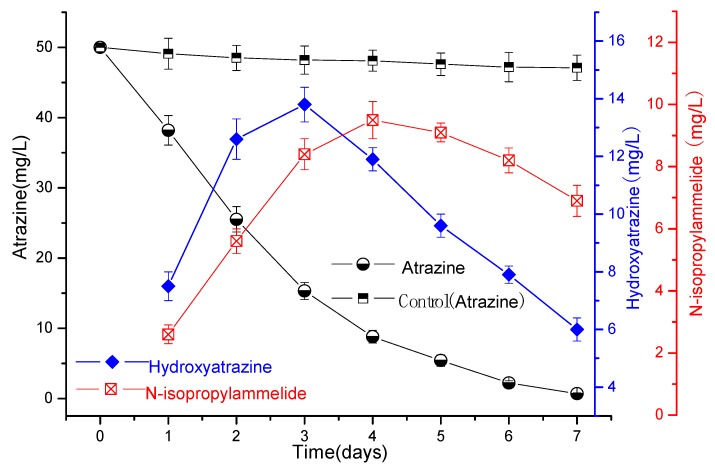
The curve of the degradation of atrazine by the strain ATLJ-5.

**Figure 4:**
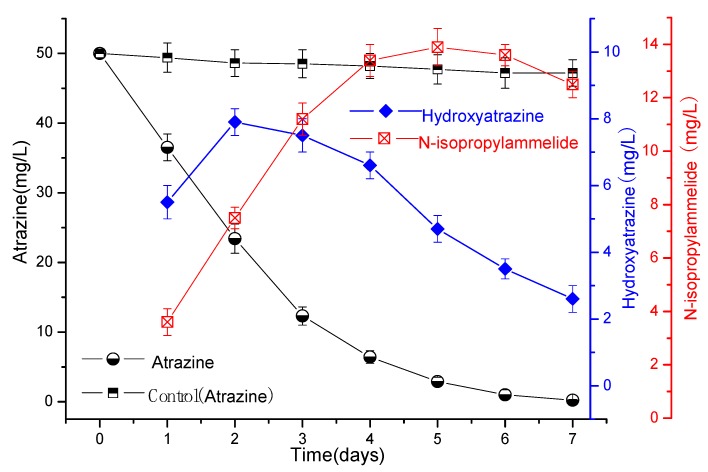
The curve of the degradation of atrazine by the strain ATLJ-11.

**Figure 5:**
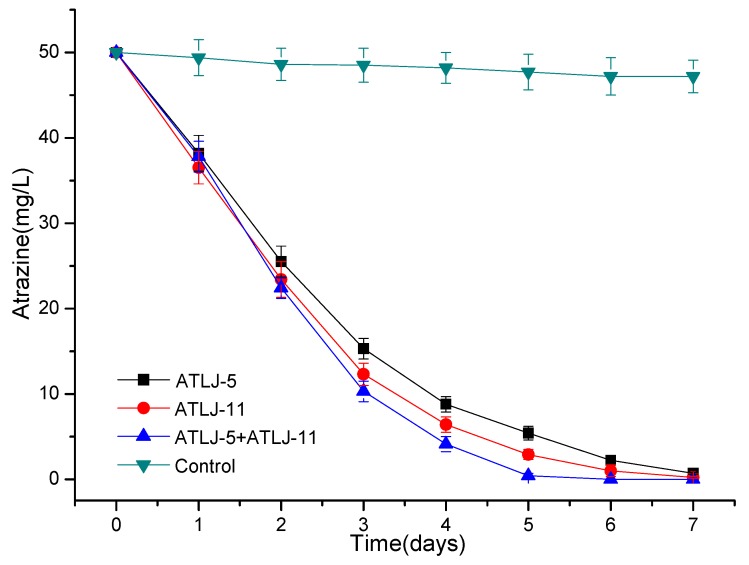
The curve of the degradation of atrazine by different microorganisms.

The results in Figure 3 and Figure 4 show that atrazine could be rapidly degraded by strain ATLJ-5 or strain ATLJ-11. The degradation efficiency of atrazine (50 mg/L) by strain ATLJ-5 can reach about 98.6% after 7 days, while that of strain ATLJ-11 can reach 99.6%. It can also be seen from Figure 5 that strain ATLJ-11 degrades atrazine faster than strain ATLJ-5. However, this does not mean that atrazine can be completely degraded into carbon dioxide and water. Hydroxyatrazine and N-isopropylammelide were important intermediate metabolites during the degradation of atrazine [10,25,26,27]. Both intermediate metabolites were detected during the degradation of atrazine by the two strains, and their concentrations both increased first and then decreased. According to the experimental data, hydroxyatrazine and N-isopropylammelide have a certain degree of accumulation during the degradation of atrazine, but both of them can continue to the degradation. Although the strain ATLJ-5 degrades hydroxyatrazine more slowly than the strain ATLJ-11, strain ATLJ-5 degrades N-isopropylammelide faster than strain ATLJ-11. Therefore, the combined application of the two strains was considered to be a better method. The experimental results in Figure 5 show that the combined application of the two strains achieved better results, and the degradation efficiency of atrazine (50 mg/L) can reach 100% after 6 days.

### 3.3. The Effect of Atrazine Concentration on the Growth of Strains ATLJ-5 and ATLJ-11

**Figure 6:**
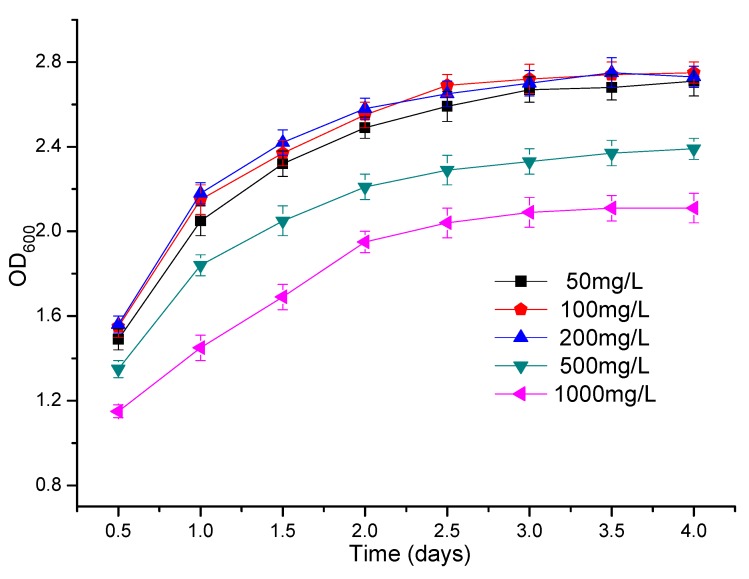
Effect of the concentration of atrazine on the growth of the strain ATLJ-5.

**Figure 7:**
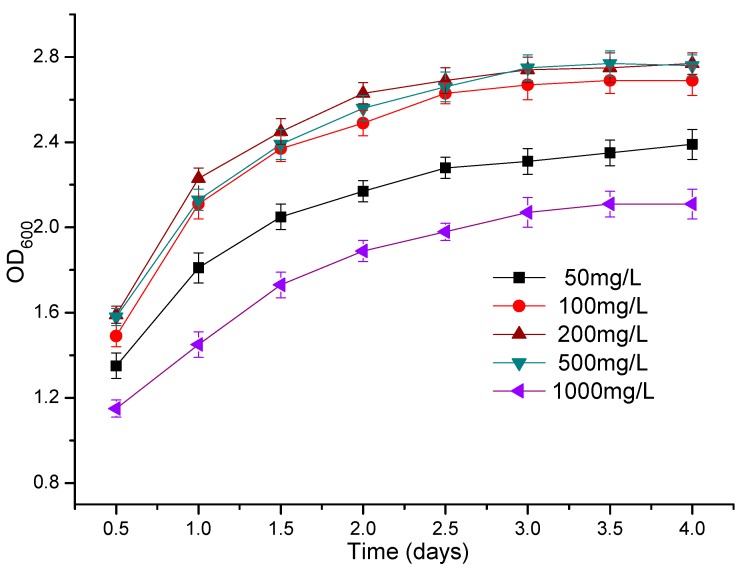
Effect of the concentration of atrazine on the growth of the strain ATLJ-11.

When the concentration of atrazine increased from 50 mg/L to 200 mg/L (Figure 6), the OD_600_ of the strain ATLJ-5 did not change significantly (*p* > 0.05). The OD_600_ would decrease if the concentration of atrazine continued to increase to 500 mg/L or 1000 mg/L (Figure 6), indicating that 500 mg/L atrazine (or a higher concentration) could significantly inhibit the growth of strain ATLJ-5. The OD_600_ of strain ATLJ-11 was at the highest level when the concentration of atrazine was 100–500 mg/L (Figure 7). A concentration of 50 mg/L of atrazine may not meet the carbon source or energy requirements for the growth of strain ATLJ-11, resulting in a decrease in the OD_600_. Although 1000 mg/L of atrazine would cause a significant decrease in the OD_600_ of strains ATLJ-5 and ATLJ-11, they could still grow. Therefore, it was preliminarily considered that strain ATLJ-5 and strain ATLJ-11 could tolerate at least 1000 mg/L of atrazine under the experimental conditions. In addition, comparing Figure 6 and Figure 7, it was found that 500 mg/L of atrazine had no effect on the growth of strain ATLJ-11 and significantly inhibited the growth of strain ATLJ-5. According to the results, it was considered that strain ATLJ-5 and strain ATLJ-11 had good atrazine degradation ability, which could be used in a wider range of atrazine concentrations. In addition, it was possible that they could be used in the bioremediation of atrazine pollution.

### 3.4. Degradation of Atrazine by a Simple Microbial Consortium or Microbial Agent

**Figure 8:**
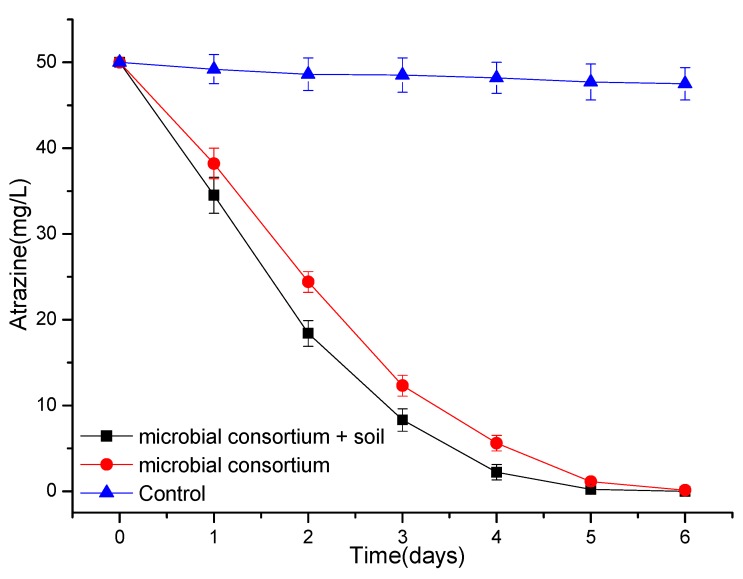
The curve of the degradation of atrazine by a microbial consortium and natural soil.

**Figure 9:**
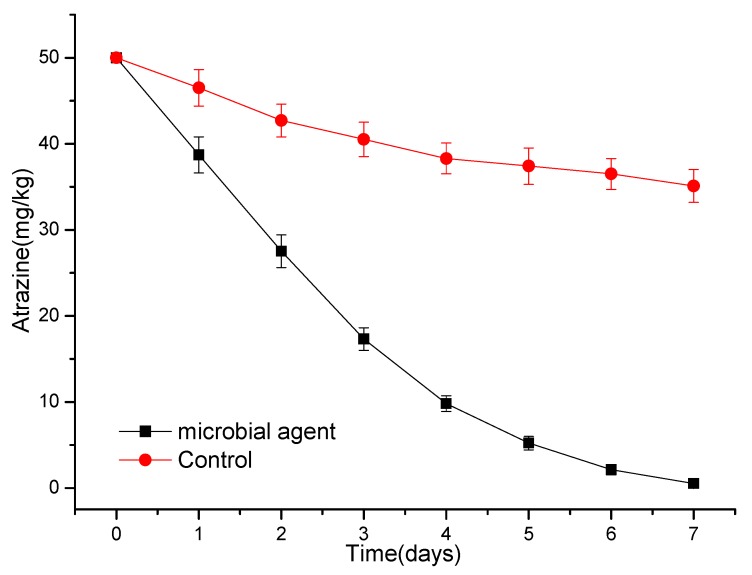
The curve of the degradation of atrazine in soil by a microbial agent.

A simple microbial consortium was constructed by using strain ATLJ-5 the strain ATLJ-11, and the effect of this microbial consortium on the degradation of atrazine was tested. The degradation efficiency of atrazine (50 mg/L) by a microbial consortium can reach about 99.8% after 6 days (Figure 8). When the microbial consortium was mixed with soil for the degradation of atrazine, its degradation efficiency could be significantly improved. Therefore, when preparing a microbial agent, adding an appropriate amount of soil may be an effective means for improving the degradation effect. The results of using the microbial agent prepared in this study to degrade atrazine residues in soil are shown in Figure 9. The degradation efficiency of atrazine (50 mg/kg) could reach 99.0% by the microbial agent after 7 days. The survival of bioaugmentation strains was a key problem in the process of microbial degradation [28,29]. The strain ATLJ-5 and the strain ATLJ-11 were speculated to survive well in both soil or a microbial agent because the microbial agent of two strains could degrade atrazine in soil effectively. In the control group without a microbial agent (Figure 9), the degradation efficiency of atrazine was 29.8% after 7 days, which was attributed to the action of natural microbes, degrading enzymes, and other factors in the soil. Therefore, it was considered that the addition of atrazine-degrading bacteria is a powerful tool to remediate contaminated soil [30]. Although the effect of microbial agents on the treatment of atrazine residues in deep soil was not good enough, the application of microbial agents at the appropriate time after the use of atrazine would effectively reduce its transfer to deep soil [12,30,31].

## 4. Conclusions

In this study, two atrazine-degrading bacteria were isolated from soil—*B. licheniformis* ATLJ-5 and *B. megaterium* ATLJ-11. The degradation efficiency of atrazine (50 mg/L) by the strain ATLJ-5 can reach about 98.6% after 7 days, and the strain ATLJ-11 can reach 99.6% under the same conditions. The degradation of atrazine is faster when the two strains are used in combination. Adding a proper amount of fresh soil during the degradation of atrazine by these two strains can also increase the degradation efficiency. The strains ATLJ-5 and ATLJ-11 have a high tolerance to atrazine and can tolerate at least 1000 mg/L of atrazine under the experimental conditions. In addition, the strains ATLJ-5 and ATLJ-11 have been successfully made into a microbial agent that is easy to commercialize and can be used to treat atrazine residues in soil. The microbial agent containing the strain ATLJ-5 and the strain ATLJ-11 achieved good results in this experiment. The degradation efficiency of atrazine (50 mg/kg) could reach 99.0% by the microbial agent after 7 days. These results suggest that the strain ATLJ-5 and the strain ATLJ-11 can be used for the treatment of atrazine pollution.
